# Integrated Analysis of a Competing Endogenous RNA Network Revealing a Prognostic Signature for Cervical Cancer

**DOI:** 10.3389/fonc.2018.00368

**Published:** 2018-09-06

**Authors:** Leilei Xia, Han Wang, Shengyun Cai, Xiaoling Su, Jizi Shen, Qi Meng, Yu Chen, Li Li, Jiuqiong Yan, Caihong Zhang, Mingjuan Xu

**Affiliations:** ^1^Department of Obstetrics and Gynecology, Changhai Hospital, Second Military Medical University, Shanghai, China; ^2^Department of Pathology, Eastern Hepatobiliary Surgery Hospital, Second Military Medical University, Shanghai, China; ^3^Department of Obstetrics and Gynecology, No. 455 Hospital, Shanghai, China

**Keywords:** cervical cancer, ceRNA network, prognostic factor, FASN, ERG

## Abstract

Given the high morbidity and the trend of younger individuals being affected observed in cervical cancer, it is important to identify sensitive and effective biomarkers for predicting the survival outcome of patients. Based on data from 307 cervical cancer cases acquired from The Cancer Genome Atlas portal, 1920 differentially expressed mRNAs, 70 microRNAs(miRNAs), and 493 long non-coding(lncRNAs) were screened by comparing cervical cancer tissues with paracancerous tissues. A competing endogenous (ceRNA) network containing 50 lncRNAs, 16 miRNAs, and 81 mRNAs was constructed. Eighteen RNAs, comprising 13 mRNAs, 2 miRNAs, and 3 lncRNAs, were identified as significant prognostic factors by univariate Cox proportional hazards regression. ETS-related gene and fatty acid synthase signatures were discovered using a multivariate Cox regression model built to identify independent prognostic factors in cervical cancer patients. Receiver operating characteristic (ROC) analysis was used to determine the optimal cut-off value for distinguishing the risk level of cervical cancer patients. High-risk patients exhibited a poorer prognosis than low-risk patients did. This study focused on ceRNA networks to provide a novel perspective and insight into cervical cancer and suggested that the identified signature can serve as an independent prognostic biomarker in cervical cancer.

## Introduction

Cervical cancer (CC) is one of the leading malignant carcinomas threatening women's health and represents a worldwide concern (1). Since the introduction of the Papanicolaou test and human papilloma (HPV) DNA test, the incidence and mortality of CC has dramatically reduced (2). The long transmit time from cervical intraepithelial lesions to CC provides an opportunity to identify pre-cancerous lesions and enable early treatment. Once the disease develops into CC, the overall survival rate directly depends on stage, with an average survival of 70% at 5 years (3). However, approximately 11.4% of women are not screened using the effective screening systems mentioned above (4). Although HPV was identified as a driving factor that can lead to CC, a substantial proportion of CC tissues are HPV-negative (5). Given the high morbidity of this disease and the trend of younger individuals being affected (6), it is important to identify sensitive and effective biomarkers.

mRNA, microRNA(miRNA), long non-coding(lncRNA), and competing endogenous (ceRNA) represent a new RNA mechanism that regulates gene expression. Assessing miRNA regulatory networks to provide new perspectives for evaluating cancer regulatory networks is complex (7). Using miRNA as a bridge, lncRNA can modulate cell functions by regulating mRNA expression levels.

The Cancer Genome Atlas (TCGA) is a publicly available dataset that provides many types of genomic data (8). This information can be mined to construct a lncRNA-miRNA-mRNA ceRNA network in CC. In this study, we obtained RNA-seq profiles from TCGA and performed integrated analysis of differentially expressed RNAs. By using Cox regression analysis, a signature based on ETS-related gene (ERG) and fatty acid synthase (FASN) showed potential as a biomarker with prognostic value in CC.

## Methods and materials

### Data source and clinical information

High-throughput sequencing-counts (HTSeq-counts) and miRNA sequencing profiles were obtained from TCGA data portal, which included 307 CC patients as of March 1, 2018. Three profiles were obtained from normal or adjunct cervical tissues, whereas the other signatures were obtained from CC tissues.

Clinical information from CC patients was also downloaded from TCGA. Of the 307 patients, 254 patients had cervical squamous cell carcinoma and 53 patients had adenocarcinoma carcinoma. The median age of these population was 46 years. According to clinical stage, 188 individuals were stage I-IIa while 109 individuals were stage IIb-IV. According to the status after surgical treatment, 191 patients were tumor free, whereas 74 patients survived with tumors. At the time of the last follow-up, 247 patients were still alive, whereas 60 had died. Infection with HPV was not consistently reported, with only 15 patients reported to have HPV. Thus, the HPV subgroup was not analyzed.

### Identification of differentially expressed mRNAs, miRNAs, and lncRNAs

All expression profiles were normalized within and among samples. mRNA and lncRNA were classified by their HTSeq-counts based on CRCh38.87 mapping. To identify potential RNAs involved in the development of CC, differentially expressed RNAs between normal or adjunct cervical tissues and CC tissues were analyzed using the edgeR package with a cutoff of false discovery rate-adjusted *p* < 0.01 and |logFC|≥2 (9). Because many RNAs exhibit low expression, RNAs with a mean expression of less than 1 were excluded. The heatmap was plotted using the heatmap.2 function in gplots package.

### Construction of the ceRNA network

Because mRNA, miRNA, and lncRNA can regulate each other, it is necessary to construct a ceRNA network to provide insight into their interaction mechanisms. Thus, we set miRNA as the central point and differentially expressed RNAs were analyzed based on the following pairings: miRNA-lncRNA and mRNA-miRNA. Briefly, according to the miRcode database (10), differentially expressed lncRNAs were used to pair differentially expressed miRNAs. Next, the paired miRNAs were used to identify target mRNAs according to three databases: miRDB (11), miRTarBase (12), and TargetScan (13). Only mRNAs predicted by all three databases were defined as target mRNAs. Next, the intersection of target mRNAs and differentially expressed mRNAs was selected to construct the ceRNA network along with the miRNAs and lncRNAs mentioned above.

### Functional enrichment analysis of gene ontology and KEGG pathways

The intersections of target mRNAs and differentially expressed mRNAs were subjected to functional enrichment analysis, including gene ontology and KEGG pathway analysis. The mRNAs involved in the ceRNA network were divided into two groups: upregulated mRNAs and downregulated mRNAs. The clusterProfiler package was utilized to analyze function enrichment (14). A *p* < 0.05 was considered to indicate significant enrichment.

### Construction of a prognostic signature based on the ceRNA network

Univariate Cox proportional hazards regression analyses were performed based on mRNAs, miRNAs, and lncRNAs in the ceRNA network. Prognosis-related RNAs were defined using the cutoff of *p* < 0.05. Next, a multivariate Cox proportional hazards regression model was constructed based on RNAs with a *p* < 0.01 to predict the risk score of each patient based on the expression of RNA. The risk score for predicting overall survival was calculated as follows: exp_RNA1_*β_RNA1_+ exp_RNA2_*β_RNA2_+ exp_RNA3_*β_RNA3_+…exp_RNAn_*β_RNAn_, where exp_RNA_ is the expression level of RNA and β_RNA_ is the regression coefficient calculated by the multivariate Cox proportional hazards regression model. Patients were divided into a low-risk group and high-risk group according to the mean risk score. Kaplan-Meier curve analysis was conducted to compare the survival times of the low-risk group and high-risk group. *P* < 0.05 was considered statistically significant. Chi-square test was utilized to correlate risk level with clinical parameters including age, race, body mass index (BMI), menopause status, histological type, tumor grade, clinical stage, neoplasm status, and vital status. Receiver operating characteristic (ROC) curve analysis using 3 years as the predicted time was also performed to estimate the predictive value of the outcome. The area under the ROC curve along with sensitive and specificity were used as evaluation indices of the prediction value.

A Cox proportional hazards regression model was further used to better evaluate the effects of clinical characteristics and risk score on the overall survival of CC patients.

### Protein-protein interaction network construction

The protein-protein interaction network was constructed using the STRING website (15), which is a tool for analyzing the relationship between proteins and scoring these relationships based on experimental data and forecast data. The interaction network was visualized using Cytoscape software.

### Statistical analysis

In the analysis of differently expressed genes, significance was tested using the optimal discovery procedure and generalized likelihood ratio tests. *p* < 0.01 and |logFC|≥2 were considered statistically significant.

Enumeration data were described by sample size and constituent ratio and chi-square test was utilized correlate the risk level with clinical parameters. A univariate Cox proportional hazards model was used to assess the risk factors associated with the outcomes of CC patients, and then clinical parameters with *p* < 0.05 were entered in multivariate Cox proportional hazards model to evaluate prognostic factors. Kaplan-Meier plot analysis was used to determine the overall survival rate and the Log-rank test was utilized to analyze the differences between groups. The ROC curve was applied to calculate the sensitivity and specificity of risk score. *P* < 0.05 was considered statistically significant.

## Results

### Identification of differentially expressed mRNAs, miRNAs, and lncRNAs

A total of 19676 mRNAs, 1881 miRNAs, and 14487 lncRNAs were extracted from TCGA dataset. Differentially expressed RNAs were analyzed using the edgeR package following extraction of the expression matrix from 307 CC cases. Using *p* < 0.05 and |logFC|≥2 as the cut-off criteria, 1920 differentially expressed mRNAs (712 upregulated and 1208 downregulated), 70 differentially expressed miRNAs (33 upregulated and 37 downregulated), and 493 differentially expressed lncRNAs (128 upregulated and 365 downregulated) were identified by comparing CC and adjacent normal cervical tissues (Supplement Figure 1).

### Construction of the ceRNA network based on predicted miRNA targets

**Figure 1:**
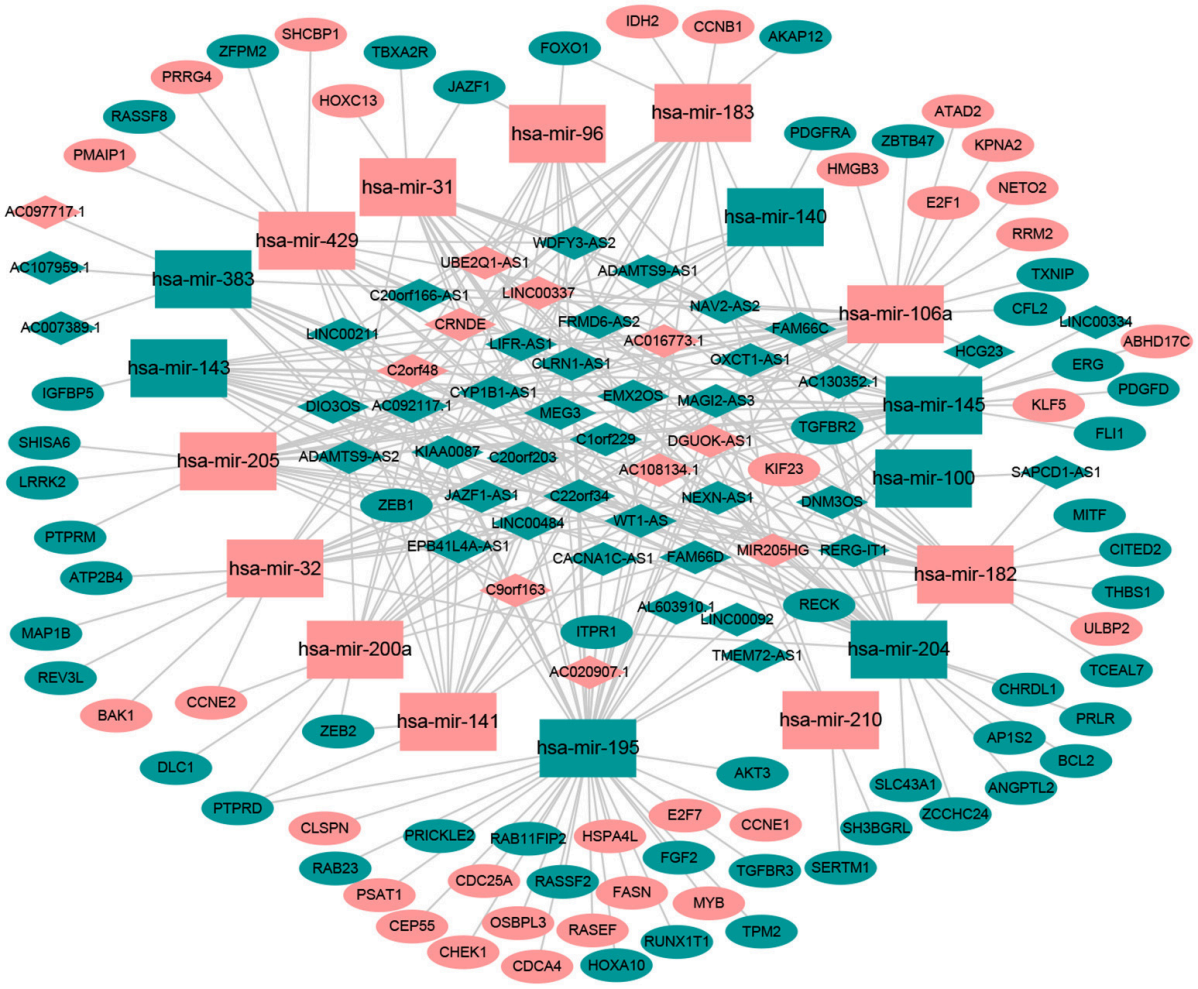
The ceRNA network of lncRNA-miRNA-mRNA. Green diamonds represent downregulated lncRNA, red diamonds indicate upregulated lncRNA, green rectangles indicate downregulated miRNA, red rectangles indicate upregulated miRNA, green ellipses indicate downregulated mRNA, and red ellipses indicate upregulated mRNA.

Numerous studies have suggested that lncRNAs interact with miRNA response elements to act as miRNA sponges (16, 17). First, the target regulation network of lncRNA-miRNA was constructed. Using miRcode to screen for potential miRNA response elements of lncRNA, 242 potential lncRNA-miRNA pairs, including 50 lncRNAs and 18 miRNAs, were formed (Supplement Table 1). Next, the miRNA-mRNA target regulation network was further assessed using miRDB, TargetScan, and miRTarBase. In total, 98 miRNA-mRNA pairs were identified, including 81 mRNAs and 16 miRNAs (Supplement Table 2). The lncRNA-miRNA-mRNA network was then constructed using Cytoscape software based on the results above and contained 50 lncRNAs, 16 miRNAs, and 81 mRNAs (Figure 1).

### Enrichment analysis of gene ontology and KEGG pathways

**Figure 2:**
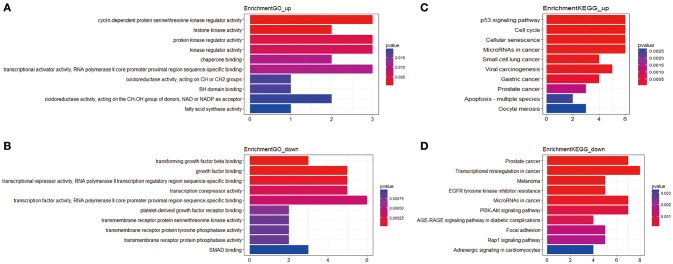
The functional enrichment analysis of mRNAs in the ceRNA network. **(A)** Gene Ontology enrichment analysis of upregulated mRNAs. **(B)** Gene Ontology enrichment analysis of downregulated mRNAs. **(C)** KEGG pathway analysis of upregulated mRNAs. **(D)** KEGG pathway analysis of downregulated mRNAs. The horizontal axis represents gene counts. The vertical axis represents enrichment analysis terms.

In total, 81 mRNAs were involved in the ceRNA network: 31 upregulated mRNAs and 50 downregulated mRNAs. ClusterProfiler package was utilized to compare gene clusters based on their enriched biological processes with a cutoff of *p* < 0.05. In GO functional enrichment analysis, the upregulated mRNAs that were significantly enriched included cyclin-dependent protein serine/threonine kinase regulator activity, histone kinase activity, protein kinase regulator activity, kinase regulator activity, and chaperone binding. The downregulated mRNAs that were significantly enriched included transforming growth factor beta binding, growth factor binding, transcriptional repressor activity, RNA polymerase II transcription regulatory region sequence-specific binding, transcription corepressor activity, transcription factor activity, and RNA polymerase II core promoter proximal region sequence-specific binding (Supplement Table 3). In the KEGG analysis, the upregulated mRNAs were mainly enriched in p53 signaling pathway, cell cycle, cellular senescence, microRNAs in cancer, and small cell lung cancer. Downregulated mRNAs were mainly enriched in prostate cancer, transcriptional misregulation in cancer, melanoma, epidermal growth factor receptor (EGFR) tyrosine kinase inhibitor resistance, and microRNAs in cancer (Supplement Table 4) (Figure 2).

### Construction of a prognostic signature based on the ceRNA network

**Figure 3:**
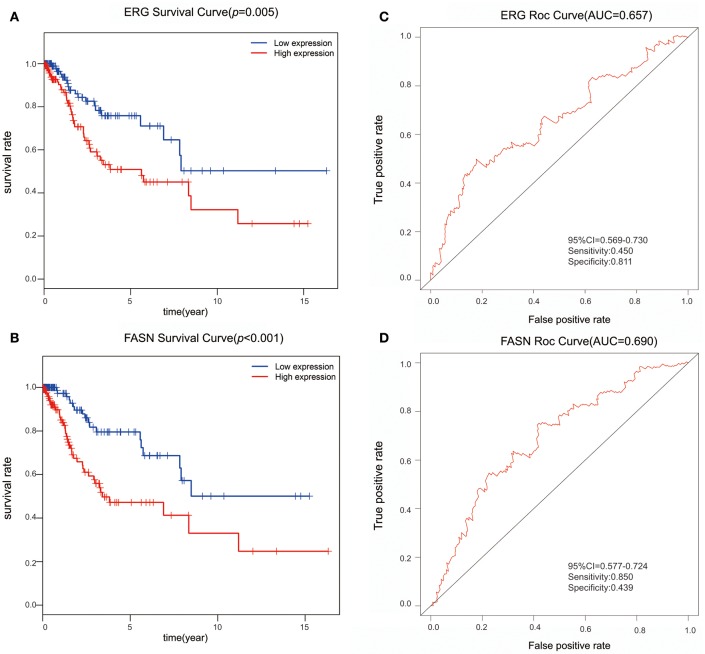
The predictive value of ERG and FASN on outcome. **(A)** The Kaplan-Meier survival analysis of ERG expression in overall survival. **(B)** The Kaplan-Meier survival analysis of FASN expression in overall survival. **(C)** ROC cure for predicting 3-year survival based on ERG expression levels. **(D)** ROC curve for predicting 3-year survival based on FASN expression level.

**Figure 4:**
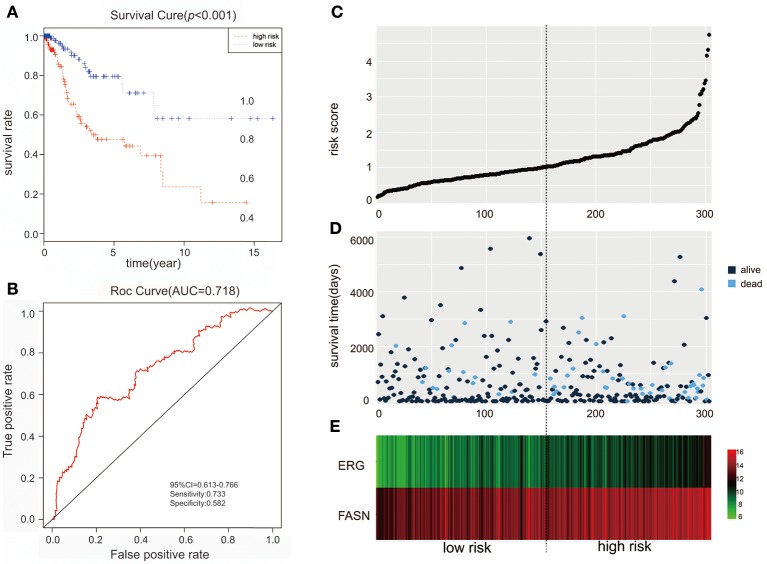
The predictive value of the risk score calculated by ERG and FASN on outcome. **(A)** Kaplan-Meier survival analysis of the risk score for overall survival. **(B)** ROC curve for predicting 3-year survival based on risk score. **(C)** Risk score of each individual. **(D)** Survival status and survival time of each individual. **(E)** Heatmap of ERG and FASN in high-risk and low-risk groups.

We further performed survival and prognostic analyses of the 50 lncRNAs, 16 miRNAs, and 81 mRNAs in the ceRNA network. Univariate Cox proportional hazards regression revealed that 18 RNAs, comprised of 13 mRNAs, 2 miRNAs, and 3 lncRNAs, were identified as significant prognostic factors based on the cutoff of a *p* < 0.05 (Supplement Table 5). Multivariate Cox proportional hazards regression analysis revealed that two RNAs exhibited a significant prognostic value: ERG and FASN (Supplement Table 6) (Figure 3). The risk assessment score for predicting overall survival was calculated as follows: Risk score = exp_ERG_*0.359+ exp_FASN_*0.345. Based on the risk score, cervical cancer patients were divided into two group: low-risk and high-risk. Kaplan-Meier survival analysis indicated that the mean survival time was 2154 days for the high-risk group and 4166 days for the low-risk group (*p* < 0.001). In ROC curve analysis, the 3-year survival of the area under the curve was 0.718 (*p* < 0.001, 95% confidence interval of hazard ratio: 0.613-0.766, sensitivity: 0.733, specificity: 0.582) (Figure 4).

The clinical parameters along with risk level are shown in Table 1. In the Chi-square test, as shown in Table 2, the risk level was significantly related to BMI (*p* = 0.006), histological type (*p* < 0.001), clinical stage (*p* = 0.001), and vital status (*p* < 0.001). No dramatic correlation between the risk level and other clinical parameters such as age, race, or neoplasm status were found.

**Table 1:** Clinical parameters of cervical cancer patients.

**Subgroup**	**Frequency**	**Percent**	**Valid percent**
Age			
<60	238	78.6	78.6
>=60	65	21.4	21.4
Race			
White	209	68.8	91.3
Asian	20	6.6	8.7
BMI			
<25	98	32.2	37.8
>=25	161	53	62.2
Menopause			
Premenopausal	124	40.8	53.7
Perimenopause/postmenopausal	107	35.2	46.3
Histological type			
Squamous cellcarcinoma	252	82.9	82.9
Adenocarcinoma/adenosquamous carcinoma	52	17.1	17.1
Grade			
G1+G2	153	50.3	56.3
G3	119	39.1	43.8
Clinical stage			
I-IIA2	188	61.8	63.3
IIB-IV	109	35.9	36.7
Neoplasm status			
Tumor free	188	61.8	71.8
With tumor	74	24.3	28.2
Vital status			
Alive	244	80.3	80.3
Dead	60	19.7	19.7
Risk level			
Low	152	50	50
High	152	50	50

**Table 2:** Relationship between risk level and clinical parameters.

**Subgroup**	**High-risk**	**Low-risk**	**Total**	***P*-value**
Age				0.484
<60	122	117	239	
>=60	30	35	65	
Race				0.684
White	105	104	209	
Asian	11	9	20	
BMI				0.006^*^
<25	58	40	98	
>=25	67	94	161	
Menopause				0.315
Premenopausal	65	59	124	
Perimenopause/postmenopausal	49	58	107	
Histological type				<0.001^*^
Squamous cellcarcinoma	139	113	252	
Adenocarcinoma/adenosquamous carcinoma	13	39	52	
Grade				0.783
G1+G2	72	81	153	
G3	58	61	119	
Clinical stage				0.001^*^
I-IIA2	80	108	188	
IIB-IV	68	41	109	
Neoplasm statue				0.17
Tumor free	89	99	188	
With tumor	42	32	74	
Vital status				<0.001^*^
Alive	108	136	244	
Dead	44	16	60	

* *p < 0.05*.

**Figure 5:**
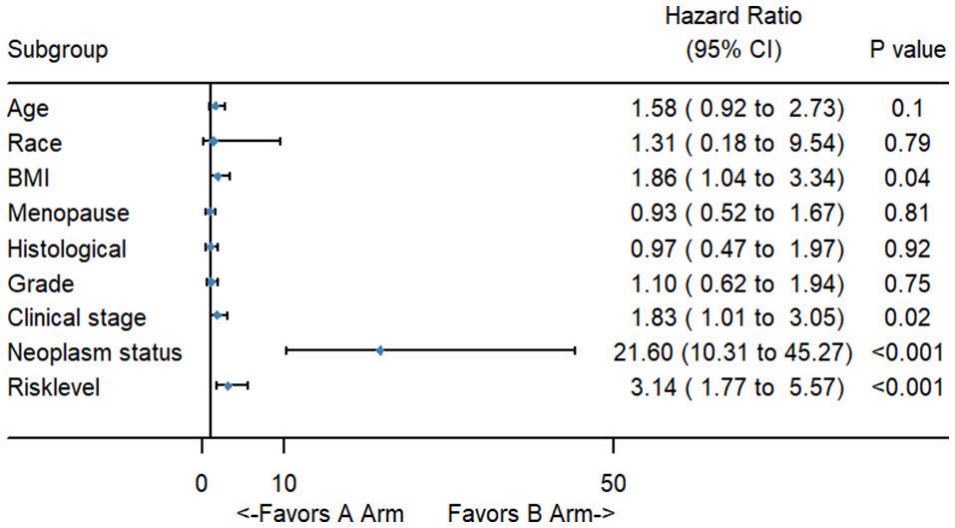
The forest map of univariate logistic regression analysis. The line provided an imaginal of 95% CI, location of diamond on the line represented the odds ratio.

**Figure 6:**
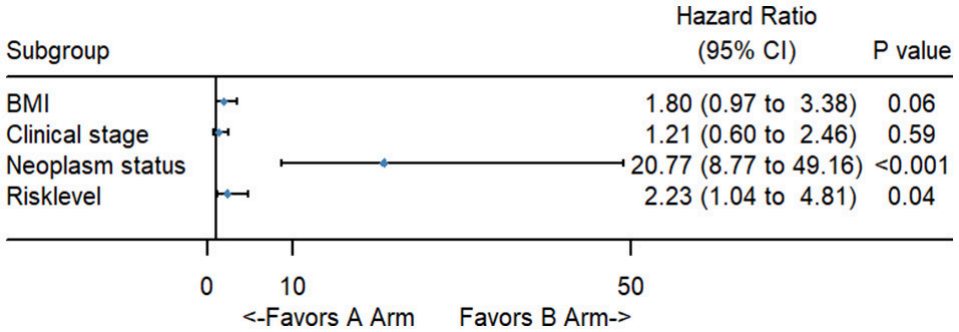
The forest map of multivariate logistic regression analysis. The line provided an imaginal of 95% CI, location of diamond on the line represented the odds ratio.

To better evaluate the prognostic value of the risk score, clinical characteristics, including race, age, tumor grade, clinical stage, pathological type, type of neoplasm, BMI, and menopause status, were examined in survival analysis. Univariate analysis of clinicopathological parameters revealed that survival time was significantly associated with clinical stage (*p* = 0.02), type of neoplasm (*p* < 0.001), BMI (*p* = 0.04), and risk score (*p* < 0.001). However, no significant differences were found between the survival time and race, age, tumor grade, pathological type or menopause status (Figure 5). Unexpectedly, multivariate Cox proportional hazards regression analysis indicated that type of neoplasm (*p* < 0.001) and risk level (*p* = 0.04) were significantly independent predictive factors of poorer prognosis for CC but not clinical stage (*p* > 0.05) (Figure 6).

### Protein-protein network analysis

**Figure 7:**
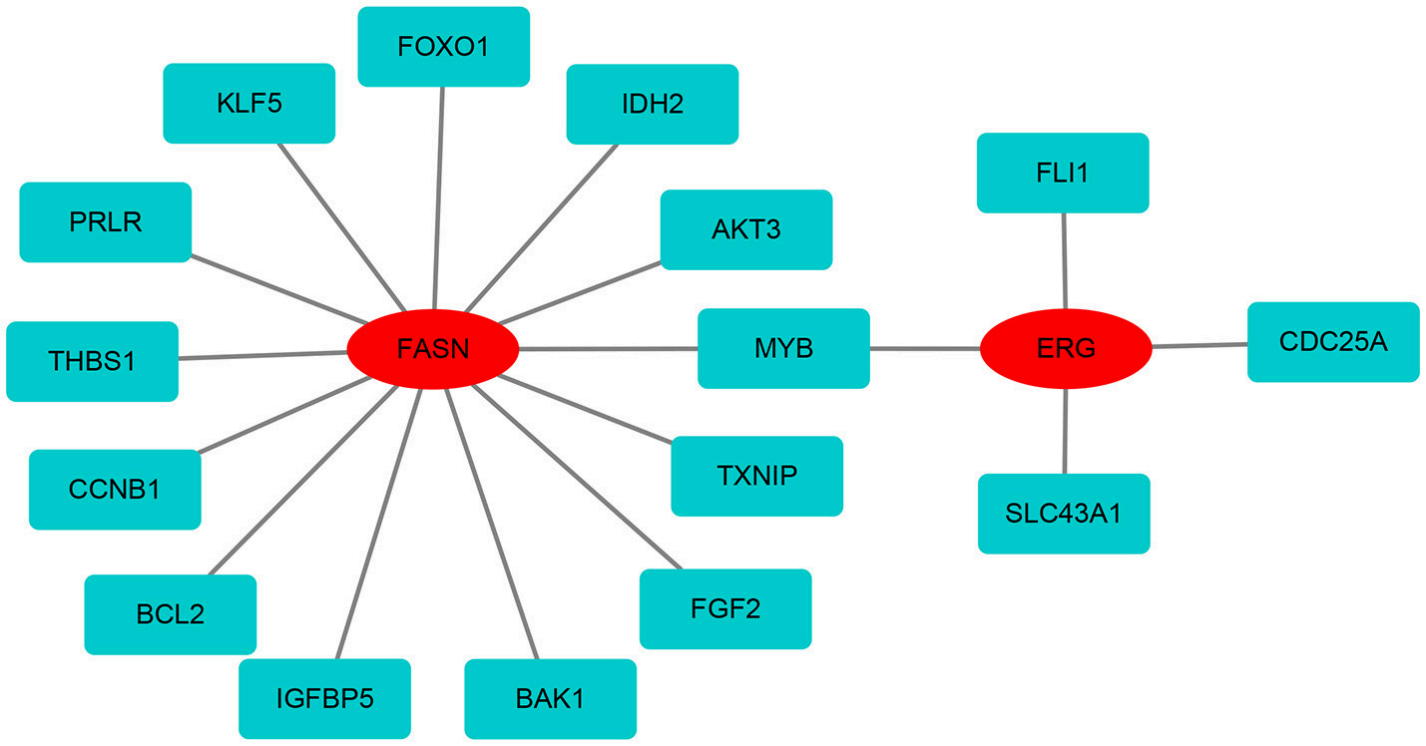
The protein-protein network of FASN and ERG with differentially expressed mRNAs. FASN and ERG were surrounded by differentially expressed mRNAs based on the STRING database.

Based on the differentially expressed mRNAs, the protein-protein network was constructed using FASN and ERG as the center. We found that FASN was related to FOXO1, KLF5, PRLR, THBS1, CCNB1, BCL2, IGFBP5, BAK1, FGF2, TXNIP, MYB, AKT3, and IDH2, whereas ERG was closely related to FLI1, CDC25A, SLC43A1, and MYB (Figure 7).

## Discussion

Screening systems such as the Pap and HPV DNA test dramatically reduced the incidence and morality of CC, but its high sensitivity and low specificity contribute to colposcopy in numerous women with doubtful lesions. Increasing studies have performed to identify biomarkers to distinguish disease stages and some progress has been made. P16ink4a, p16, E-cadherin, Ki67, pRb, and p53 are markers that can discriminate CC from intraepithelial lesions, while CEA, SCC, and CD44 are used to detect invasive cancer (3). Among them, P16ink4a is directly linked to HPV oncogenic action, and is overexpressed in nearly all cases of high-grade squamous intraepithelial lesion but rarely in low-grade squamous intraepithelial lesion (18). P16ink4a and Ki67 can improve the sensitivity and specificity of the equivocal atypical squamous cells of undetermined significance or low-grade squamous intraepithelial lesion Pap test results, reducing the requirement for colposcopy.

In this study, differentially expressed RNAs, including mRNA, miRNA, and lncRNA, were identified by comparing adjunct or normal cervical tissues with CC tissues. Using miRNA as a bridge, paired lncRNA-miRNA-mRNAs were screened to construct a ceRNA network. To explore the effect of the ceRNA network on survival status, univariate and multivariate Cox proportional hazard regression analyses were conducted to successively analyze the prognostic value of differentially expressed RNAs. According to the expression level of prognostic genes, CC patients were divided into low-risk and high-risk groups. Next, clinical characteristics and risk scores were analyzed. TNM stage, BMI, type of neoplasm, and risk score were identified as prognostic factors of CC patients.

Compared to mRNA, lncRNA was recently discovered. Abundant evidence indicates that lncRNAs interact with the miRNA response element and function as miRNA sponges. Using miRNA as a bridge, ceRNA networks involving mRNA and lncRNA have been constructed, providing insight into CC. Previous studies revealed interactions between miRNA and mRNA. Functional analysis of differentially expressed miRNA targets revealed numerous cancer-associated pathways, cell growth and death-related pathways, including apoptosis, cell cycle, p53 signaling pathway and other signal transduction pathways, such as ErbB, MAPK, mTOR, Notch, TGF-β, and Wnt. Particularly, based on bioinformatics analysis of datasets including GEO and TCGA, key genes have been identified and found to be involved in cell proliferation including TP53, MYC, CCND1, CDKN2A, JUN, FOS, which are targets of differentially expressed miRNAs in CC. These transcription regulators may form feedback loops with miRNAs to modulate cell apoptosis and migration in CC. A pioneer study based on the interaction of mRNA and miRNA indicated that CHEK1, SOX17, CDKN2A, and CEP55, which are targets of star miRNAs in CC including miR-424-5p, miR-200a-5p, miR-9-5p, and miR-133b, play a vital role in the development of CC (19). However, the combination of lncRNA, miRNA and mRNA expression profile has not widely examined in CC. In this study of ceRNA, 50 lncRNAs, 16 miRNAs, and 81 mRNAs were found to be involved in cervical carcinogenesis.

miRNA are short regulatory RNAs that modulate gene expression levels by partial base pairing with the 3′ untranslated region of their target mRNAs. miRNAs show partial nucleotide sequence paring between miRNAs and their mRNA targets, leading to promiscuous interactions: one miRNA may interact with one or more mRNAs and one mRNA may be targeted by multiple miRNAs. miRNAs play vital roles in maintaining physiological function, including development, metabolism, senile, and apoptosis. Dysregulation of miRNAs can result in various diseases.

In the progression of CC, miRNAs were analyzed according to the severity of disease including CIN1, CIN2, CIN3, and CC. Few changes were observed in the miRNA expression in CIN1 compared to in normal cervical tissue. As the disease progressed, increasing number of miRNAs was found to be greatly altered (20).

In preeclampsia (PE), the perturbation of angiogenesis is recognized as a vital pathogenic factor. Various studies demonstrated that miR-16, miR-26b, miR-29b, miR-335, miR-222, miR-181a, and miR-195, which are associated with vascular endothelial growth factor, were aberrantly upregulated in PE (21). Among them, miR-181a was over-expressed in both the placenta and maternal sera. Additionally, miR-181, acting as modulator of T-cell sensitivity and is associated with tolerance induction at the maternal-fetal interface, was disturbed during PE (22).

Placental vascular alterations due to angiogenic imbalance occurred in PE as well as fetal growth restriction (FGR). FGR is the termination or decrease of the fetus determined by generic factors during pregnancy. Accumulating evidence demonstrated that the dysregulation of miRNAs is involved in FGR. In placental tissue, miR-21, miR-499a-5p, miR-1-3p, miR-424, miR-141, and miR-519a are overexpressed in FGR while miR-194 are downregulated (23). In maternal circulation, miR-100-5p, miR-125-5p, and miR-199a-5p are downregulated in FGR as well as preeclampsia and gestational hypertension (24). *In vitro* and *in vivo* experiments showed that, miR-141-3p and miR-200A-3p are vital for placental development in mouse by regulating the level of insulin like growth factor 2 (25).

The survival outcome of CC is mainly predicted by two elements, TNM stage and type of neoplasm; however, these indices are not quantifiable. Some studies suggested that hypoxia-inducible factor-1α can serve as an independent poor survival marker of CC, as it is involved in numerous pathways, including the PI3K/AKT and mTOR pathways (26). Additionally, hypoxia-inducible factor-1α may be induced by HPV to promote tumor angiogenesis (27). BCL-2, a key protein in the process of transmitting apoptosis signals, is negatively correlated with disease-free survival in CC and can function as an independent survival factor (28). Cyclooxygenase-2, a cyclooxygenase subtype, can inhibit the apoptosis signal, increase vascularization, and activate the PI3K/AKT pathway to promote chemoresistance (29). Additionally, EGFR was upregulated with the degree of malignancy in CC. EGFR exhibits a positive correlation with lymph node metastasis (30). However, an efficient and sensitive marker for predicting the outcome of CC is lacking. In this study, we identified a signature based on ERG and FASN to predict the risk score of each patient and evaluate the outcome in combination with information regarding TNM stage and type of neoplasm in cervical cancer patients.

ERG is a member of the erythroblast transformation-specific family that regulates embryonic development, cell proliferation, apoptosis, and angiogenesis. It is indispensable for inducing vascular cell remodeling (31). Under normal circumstances, ERG is highly expressed in the embryonic mesoderm and endothelium. During vascular development, its expression decreases, but the pluripotency of hematopoietic stem cells continues to be regulated (32). An increasing number of studies has indicated that ERG functions in malignant cancers. In acute myeloid leukemia, ERG expression is high and predicts adverse clinical outcome (33). Several studies demonstrated that ERG is highly and consistently expressed in many prostate cancer patients because of gene fusion with TMPRSS2, an androgen-dependent gene (34). ERG serves as an actuation factor in the development of prostate cancer. ERG overexpression is associated with advanced TNM stage, high Gleason score, and poor survival outcome (35). ERG overexpression influences the epithelial-mesenchymal transition by upregulating FZD4 and downregulating E-cadherin (36). Additionally, NOTCH and Wnt signaling are activated by high levels of ERG to induce cell migration and invasion. ERG is upregulated in central nervous system tumors, including glioblastomas and hemangioblastomas, and may function as a specific marker in these tumors (37).

FASN encodes an enzyme that catalyzes the conversion of acetyl-CoA and malonyl-CoA into long-chain saturated fatty acids. FASN overexpression is associated with malignant tumors, including breast cancer (38), prostate cancer (39), colorectal cancer (40), gastric cancer (41), and lung cancer (42), as well as poor prognosis. Loss of FASN can reduce the energy metabolism, proliferation, and migration of cancer cells. FASN also inhibits the expression of HER2 and may function as a potential therapeutic target in estrogen receptor- and progesterone receptor-positive endometrial cancers (43).

Few studies have focused on the role and mechanism of ERG and FASN in CC. In our protein-protein interaction network analysis, we found that FASN and ERG proteins interact with other proteins, including FOXO1, KLF5, THBS1, CCNB1, BCL2, AKT3, and CDC25A, to play a crucial role in CC. We identified a signature based on ERG and FASN as a biomarker in CC. This signature may serve as a therapeutic target for precision medicine in CC. This study describes a new method for further investigating the pathogenesis of CC. Further studies are required to explore the biological functions and underlying molecular mechanisms of ERG and FASN in cervical cancer.

## Conclusion

This study focused on ceRNA networks to provide a novel perspective and insight into CC and suggested that the signature based on ERG and FASN can serve as an independent prognostic biomarker in CC.

## Ethics statement

High-throughput sequencing-counts (HTSeq-counts) and miRNA sequencing profiles were obtained from the TCGA data portal, which is a publicly available dataset for which no ethics approval is needed.

## Author contributions

LX constructed the ceRNA network using R software. HW analyzed the data using SPSS. SC download data from TCGA. XS used photoshop and illustration software. JS organized the data. QM performed GO and KEGG analysis. YC performed PPI network analysis. LL wrote the manuscript. JY performed survival analysis. CZ polished the language. MX provided guidance.

### Conflict of interest statement

The authors declare that the research was conducted in the absence of any commercial or financial relationships that could be construed as a potential conflict of interest.

## Supplementary material

Supplement Figure 1 Heatmap of differentially expressed RNAs. **(A)** The heatmap of 200 randomly selected differentially expressed mRNAs. **(B)** The heatmap of 70 differentially expressed miRNAs. **(C)** The heatmap of 200 randomly selected differentially expressed lncRNAs. Red indicates a high level of RNA expression, whereas green indicates low expression.

Supplement Table 1 Paired lncRNA and miRNA. 50 differently expressed lncRNAs and 18 differently expressed miRNAs paired according to the miRcode database.

Supplement Table 2 Paired miRNA and mRNA. 98 differently expressed miRNA-mRNA pairs were identified including 81 mRNAs and 16miRNAs based on the three databases: miRDB, miRTarBase, and TargetScan. Only the mRNAs predicted by all the above three databases defined as the target mRNAs.

Supplement Table 3 Gene ontology function enrichment analysis.

Supplement Table 4 KEGG pathway analysis.

Supplement Table 5 The prognostic differently expressed RNAs involved in ceRNA network by univariate cox regression analysis.

Supplement Table 6 The prognostic differently expressed RNAs involved in ceRNA network by multivariate cox regression analysis. ^*^*P* < 0.05.

## Abbreviations

TCGA: The Cancer Genome Altas
CeRNA: competing endogenous RNA
ERG: ETS Transcription Factor
FASN: Fatty Acid Synthase
ROC: receiver operating characteristic curve
HPV: Human Papillomavirus
HTSeq: high-throughput sequencing
GO: gene ontology
KEGG: Kyoto Encyclopedia of Genes and Genomes
EGFR: Epidermal Growth Factor Receptor
BMI: Body Mass Index
FOXO1: Forkhead Box O1
KLF5: Kruppel Like Factor 5
PRLR: Prolactin Receptor
THBS1: Thrombospondin 1
CCNB1: Cyclin B1
BCL2: B-Cell CLL/Lymphoma 2
IGFBP5: Insulin Like Growth Factor Binding Protein 5
BAK1: BCL2 Antagonist/Killer 1
FGF2: Fibroblast Growth Factor 2
TXNIP: Thioredoxin Interacting Protein
MYB: MYB Proto-Oncogene
AKT3: AKT Serine/Threonine Kinase 3
IDH2, Isocitrate Dehydrogenase [NADP(+)] 2: Mitochondrial
FLI1: Friend Leukemia Virus Integration 1
CDC25A: Cell Division Cycle 25A
SLC43A1: Solute Carrier Family 43 Member 1
MYB: MYB Proto-Oncogene
mTOR: Mechanistic Target of Rapamycin Kinase
HIF1-1α: Hypoxia Inducible Factor 1 Alpha Subunit
BCL2: B-Cell CLL/Lymphoma 2
COX-2: Mitochondrially Encoded Cytochrome C Oxidase II
ETS: erythroblast transformation-specific
TMPRSS2, Transmembrane Protease: Serine 2
FZD4: Frizzled Class Receptor 4
CNS: central nervous system
PPI: Protein protein interactions.
