# Supplementary figures and images for: Integrated Analysis of a Competing Endogenous RNA Network Revealing a Prognostic Signature for Cervical Cancer
Source: Front Oncol. 2018 Sep 6;8:368. doi: 10.3389/fonc.2018.00368 (PMC6135876; doi:10.3389/fonc.2018.00368)

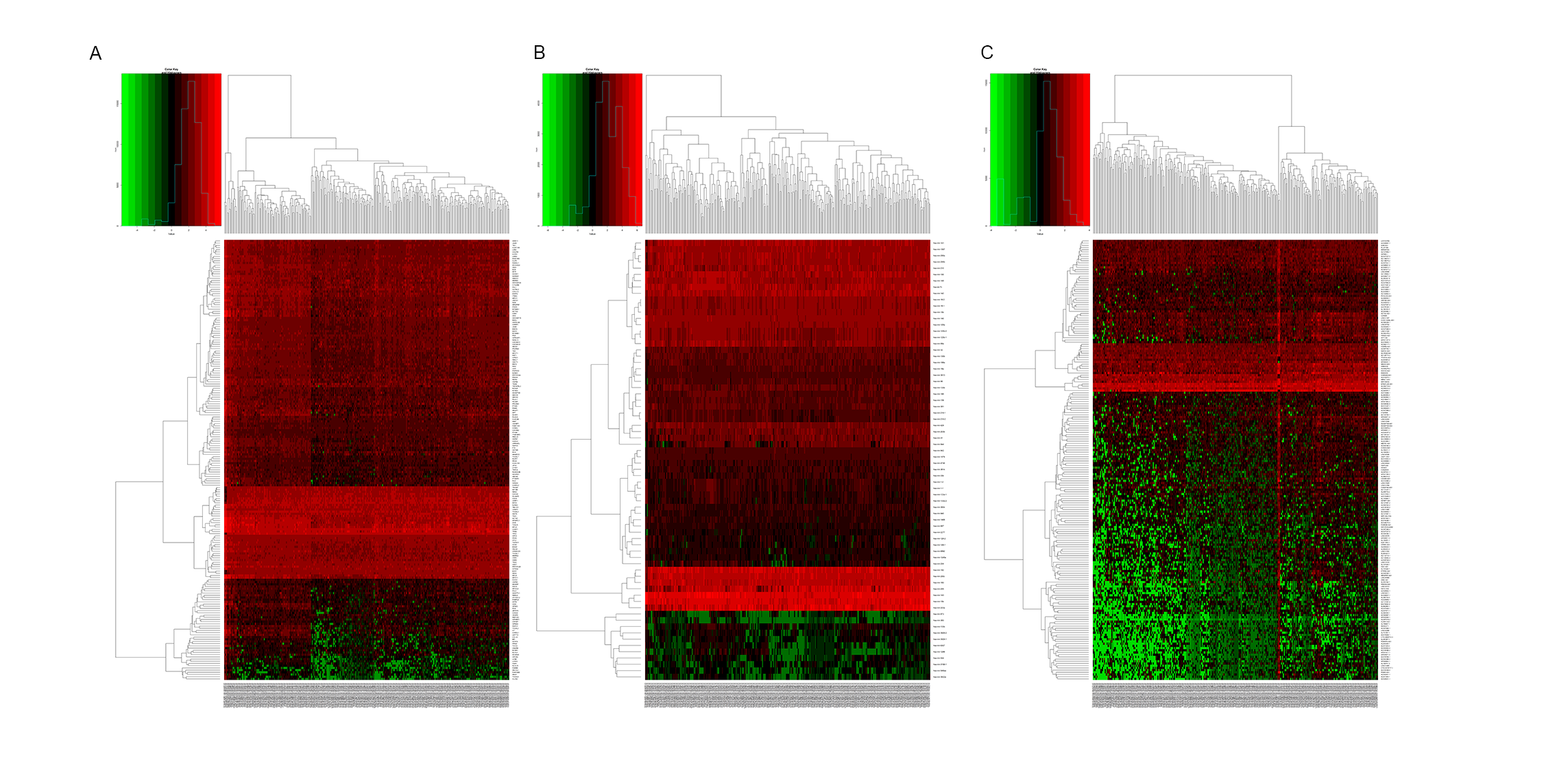

Supplement: Supplement Figure 1 — Heatmap of differentially expressed RNAs. (A) The heatmap of 200 randomly selected differentially expressed mRNAs. (B) The heatmap of 70 differentially expressed miRNAs. (C) The heatmap of 200 randomly selected differentially expressed lncRNAs. Red indicates a high level of RNA expression, whereas green indicates low expression. [file Image_1.TIF]
