# Supplementary material for: Integrated Analysis of a Competing Endogenous RNA Network Revealing a Prognostic Signature for Cervical Cancer
Source: Front Oncol. 2018 Sep 6;8:368. doi: 10.3389/fonc.2018.00368 (PMC6135876; doi:10.3389/fonc.2018.00368)
Supplement: Supplement Table 1 — Paired lncRNA and miRNA. 50 differently expressed lncRNAs and 18 differently expressed miRNAs paired according to the miRcode database. [file Table_1.DOCX]

Supplement Table 1: Paired lncRNA and miRNA

| miRNA | lncRNA |
| --- | --- |
| hsa-mir-96 | KIAA0087, WDFY3-AS2, WT1-AS, C22orf34, MEG3, UBE2Q1-AS1, ADAMTS9-AS1, ADAMTS9-AS2, LIFR-AS1, NAV2-AS2, FRMD6-AS2 |
| hsa-mir-429 | KIAA0087, C20orf166-AS1, WDFY3-AS2, WT1-AS, MEG3, EPB41L4A-AS1, FAM66C, CYP1B1-AS1, MAGI2-AS3, CLRN1-AS1 |
| hsa-mir-383 | KIAA0087, WT1-AS, C22orf34, MEG3, EPB41L4A-AS1, LINC00337, MIR205HG, LINC00211, AC107959.1, AC097717.1, AC007389.1, DIO3OS |
| hsa-mir-32 | WT1-AS, C20orf203, AC108134.1, FAM66C, JAZF1-AS1, MAGI2-AS3, LINC00484, NEXN-AS1, ADAMTS9-AS2, LIFR-AS1, CRNDE |
| hsa-mir-31 | WDFY3-AS2, WT1-AS, C22orf34, C20orf203, MEG3, AC016773.1, EMX2OS, MIR205HG, MAGI2-AS3, ADAMTS9-AS1, ADAMTS9-AS2, LIFR-AS1, CRNDE, CACNA1C-AS1, NAV2-AS2, |
| hsa-mir-210 | AC016773.1, EMX2OS, MAGI2-AS3, |
| hsa-mir-205 | C20orf166-AS1, C22orf34, C9orf163, AC130352.1, AC092117.1, MEG3, C1orf229, EPB41L4A-AS1, FAM66C, EMX2OS, MIR205HG, CYP1B1-AS1, JAZF1-AS1, CLRN1-AS1, ADAMTS9-AS2, CRNDE, CACNA1C-AS1, FAM66D, |
| hsa-mir-204 | KIAA0087, C2orf48, C22orf34, C20orf203, AC130352.1, AC092117.1, MEG3, FAM66C, UBE2Q1-AS1, DNM3OS, MIR205HG, CYP1B1-AS1, MAGI2-AS3, SAPCD1-AS1, NEXN-AS1, LINC00211, DGUOK-AS1, CLRN1-AS1, ADAMTS9-AS2, LIFR-AS1, NAV2-AS2, DIO3OS, FRMD6-AS2, |
| hsa-mir-200a | KIAA0087, WDFY3-AS2, WT1-AS, C22orf34, AC092117.1, MEG3, C1orf229, EPB41L4A-AS1, MAGI2-AS3, LINC00484, ADAMTS9-AS2, FAM66D, |
| hsa-mir-195 | KIAA0087, C2orf48, AC020907.1, WT1-AS, C22orf34, C9orf163, C20orf203, AC108134.1, AC092117.1, MEG3, AC016773.1, EPB41L4A-AS1, TMEM72-AS1, LINC00092, FAM66C, MAGI2-AS3, LINC00484, DGUOK-AS1, AL603910.1, CLRN1-AS1, CACNA1C-AS1, OXCT1-AS1, |
| hsa-mir-183 | KIAA0087, C2orf48, C20orf166-AS1, WDFY3-AS2, C20orf203, AC108134.1, EPB41L4A-AS1, FAM66C, EMX2OS, MIR205HG, LINC00211, ADAMTS9-AS2, CRNDE, |
| hsa-mir-182 | KIAA0087, WDFY3-AS2, WT1-AS, C22orf34, AC108134.1, MEG3, LINC00337, UBE2Q1-AS1, EMX2OS, ADAMTS9-AS1, ADAMTS9-AS2, LIFR-AS1, CACNA1C-AS1, NAV2-AS2, RERG-IT1, FRMD6-AS2, |
| hsa-mir-145 | KIAA0087, WDFY3-AS2, LINC00334, WT1-AS, C20orf203, AC108134.1, MEG3, AC016773.1, TMEM72-AS1, LINC00337, FAM66C, HCG23, DNM3OS, MIR205HG, CYP1B1-AS1, MAGI2-AS3, DGUOK-AS1, ADAMTS9-AS1, ADAMTS9-AS2, CRNDE, FAM66D, |
| hsa-mir-143 | C2orf48, C22orf34, C9orf163, C20orf203, AC130352.1, AC108134.1, AC092117.1, MEG3, FAM66C, EMX2OS, MIR205HG, JAZF1-AS1, MAGI2-AS3, LINC00484, ADAMTS9-AS2, CRNDE, DIO3OS, FRMD6-AS2, |
| hsa-mir-141 | KIAA0087, WDFY3-AS2, WT1-AS, C22orf34, AC092117.1, MEG3, C1orf229, EPB41L4A-AS1, MAGI2-AS3, LINC00484, ADAMTS9-AS2, FAM66D, |
| hsa-mir-140 | AC130352.1, MEG3, C1orf229, FAM66C, CLRN1-AS1, ADAMTS9-AS2, LIFR-AS1, CRNDE, |
| hsa-mir-106a | C2orf48, C20orf166-AS1, WT1-AS, AC092117.1, MEG3, EPB41L4A-AS1, LINC00337, HCG23, JAZF1-AS1, MAGI2-AS3, LINC00484, ADAMTS9-AS2, LIFR-AS1, OXCT1-AS1, DIO3OS, |
| hsa-mir-100 | AC130352.1，SAPCD1-AS1 |

50 differently expressed lncRNAs and 18 differently expressed miRNAs paired according to the miRcode database.

Supplement Table 2: Paired miRNA and mRNA

| miRNA | mRNA |
| --- | --- |
| hsa-mir-96 | ZEB1, JAZF1, FOXO1, |
| hsa-mir-429 | RASSF8, ZFPM2, ZEB1, SHCBP1, PMAIP1, PRRG4, |
| hsa-mir-32 | CCNE2, ATP2B4, REV3L, MAP1B, ITPR1, BAK1, |
| hsa-mir-31 | JAZF1, HOXC13, TBXA2R, |
| hsa-mir-210 | SERTM1, SH3BGRL, |
| hsa-mir-205 | ZEB1, LRRK2, PTPRM, SHISA6, |
| hsa-mir-204 | PRLR, ZCCHC24, ITPR1, AP1S2, TGFBR2, BCL2, CHRDL1, SLC43A1, ANGPTL2, |
| hsa-mir-200a | CCNE2, ZEB1, PTPRD, ZEB2, DLC1, |
| hsa-mir-195 | TGFBR3, MYB, FASN, PTPRD, CDC25A, CEP55, CHEK1, RUNX1T1, RASSF2, PSAT1, CLSPN, CCNE1, E2F7, TPM2, FGF2, RAB11FIP2, ITPR1, OSBPL3, CDCA4, AKT3, PRICKLE2, RAB23, HSPA4L, RASEF, RECK, HOXA10, KIF23, |
| hsa-mir-183 | AKAP12, IDH2, CCNB1, ZEB1, FOXO1 |
| hsa-mir-182 | CITED2, RECK, THBS1, TCEAL7, ULBP2, MITF |
| hsa-mir-145 | FLI1, TGFBR2, KLF5, PDGFD, ERG, ABHD17C |
| hsa-mir-143 | IGFBP5 |
| hsa-mir-141 | PTPRD, ZEB2, ZEB1 |
| hsa-mir-140 | PDGFRA, |
| hsa-mir-106a | TXNIP, KIF23, E2F1, TGFBR2, HMGB3, CFL2, NETO2, KPNA2, ATAD2, ZBTB47, RRM2 |

98 differently expressed miRNA-mRNA pairs were identified including 81 mRNAs and 16miRNAs based on the three databases: miRDB, miRTarBase, and TargetScan. Only the mRNAs predicted by all the above three databases defined as the target mRNAs.

Supplement Table 3:Gene ontology function enrichment analysis

| Term | Description | Counts | p-value |
| --- | --- | --- | --- |
| Up_regulated |  |  |  |
| GO:0016538 | cyclin-dependent protein serine/threonine kinase regulator activity | 3 | 2.16E-05 |
| GO:0035173 | histone kinase activity | 2 | 0.000431 |
| GO:0019887 | protein kinase regulator activity | 3 | 0.004365 |
| GO:0019207 | kinase regulator activity | 3 | 0.005833 |
| GO:0051087 | chaperone binding | 2 | 0.010956 |
| GO:0001077 | transcriptional activator activity, RNA polymerase II core promoter proximal region sequence-specific binding | 3 | 0.011146 |
| GO:0016725 | oxidoreductase activity, acting on CH or CH2 groups | 1 | 0.01811 |
| GO:0051400 | BH domain binding | 1 | 0.01811 |
| GO:0016616 | oxidoreductase activity, acting on the CH-OH group of donors, NAD or NADP as acceptor | 2 | 0.019212 |
| GO:0004312 | fatty acid synthase activity | 1 | 0.019904 |
| Down_regulated |  |  |  |
| GO:0050431 | transforming growth factor beta binding | 3 | 1.15E-05 |
| GO:0019838 | growth factor binding | 5 | 2.16E-05 |
| GO:0001227 | transcriptional repressor activity, RNA polymerase II transcription regulatory region sequence-specific binding | 5 | 0.00011 |
| GO:0003714 | transcription corepressor activity | 5 | 0.000291 |
| GO:0000982 | transcription factor activity, RNA polymerase II core promoter proximal region sequence-specific binding | 6 | 0.000391 |
| GO:0005161 | platelet-derived growth factor receptor binding | 2 | 0.000705 |
| GO:0004675 | transmembrane receptor protein serine/threonine kinase activity | 2 | 0.000805 |
| GO:0005001 | transmembrane receptor protein tyrosine phosphatase activity | 2 | 0.000805 |
| GO:0019198 | transmembrane receptor protein phosphatase activity | 2 | 0.000805 |
| GO:0046332 | SMAD binding | 3 | 0.000988 |

Supplement Table 4: KEGG pathway analysis

| Term | Description | Count | pvalue |
| --- | --- | --- | --- |
| Up_regulated |  |  |  |
| hsa04115 | p53 signaling pathway | 6 | 5.57E-09 |
| hsa04110 | Cell cycle | 6 | 2.12E-07 |
| hsa04218 | Cellular senescence | 6 | 9.58E-07 |
| hsa05206 | MicroRNAs in cancer | 6 | 3.56E-05 |
| hsa05222 | Small cell lung cancer | 4 | 4.95E-05 |
| hsa05203 | Viral carcinogenesis | 5 | 6.74E-05 |
| hsa05226 | Gastric cancer | 4 | 0.000309 |
| hsa05215 | Prostate cancer | 3 | 0.001308 |
| hsa04215 | Apoptosis - multiple species | 2 | 0.002527 |
| hsa04114 | Oocyte meiosis | 3 | 0.002647 |
| Down_regulated |  |  |  |
| hsa05215 | Prostate cancer | 7 | 1.40E-07 |
| hsa05202 | Transcriptional misregulation in cancer | 8 | 8.75E-07 |
| hsa05218 | Melanoma | 5 | 1.26E-05 |
| hsa01521 | EGFR tyrosine kinase inhibitor resistance | 5 | 1.98E-05 |
| hsa05206 | MicroRNAs in cancer | 7 | 0.000234 |
| hsa04151 | PI3K-Akt signaling pathway | 7 | 0.000631 |
| hsa04933 | AGE-RAGE signaling pathway in diabetic complications | 4 | 0.00082 |
| hsa04510 | Focal adhesion | 5 | 0.001506 |
| hsa04015 | Rap1 signaling pathway | 5 | 0.001755 |
| hsa04261 | Adrenergic signaling in cardiomyocytes | 4 | 0.003265 |

Supplement Table 5: the prognostic differently expressed RNAs involved in ceRNA network by univariate cox regression analysis

| gene | coefficient | HR | 95%CI of HR | *P*-value | type |
| --- | --- | --- | --- | --- | --- |
| ERG | 0.401 | 1.493 | 1.193-1.868 | 0.001* | mRNA |
| FASN | 0.391 | 1.479 | 1.157-1.891 | 0.002* | mRNA |
| BCL2 | -0.384 | 0.681 | 0.528-0.878 | 0.003* | mRNA |
| KIAA0087 | 0.229 | 1.258 | 1.067-1.483 | 0.006* | lncRNA |
| AC097717.1 | -0.294 | 0.745 | 0.595-0.932 | 0.010* | lncRNA |
| PTPRD | 0.144 | 1.154 | 1.034-1.289 | 0.011* | mRNA |
| ANGPTL2 | 0.299 | 1.348 | 1.070-1.698 | 0.011* | mRNA |
| PTPRM | 0.22 | 1.246 | 1.049-1.480 | 0.012* | mRNA |
| RAB11FIP2 | 0.657 | 1.928 | 1.141-3.261 | 0.014* | mRNA |
| hsa-mir-32 | -0.587 | 0.556 | 0.341-0.906 | 0.018* | miRNA |
| ZBTB47 | 0.51 | 1.665 | 1.074-2.584 | 0.023* | mRNA |
| hsa-mir-145 | -0.288 | 0.75 | 0.585-0.961 | 0.023* | miRNA |
| RASSF2 | -0.277 | 0.758 | 0.596-0.965 | 0.024* | mRNA |
| RECK | 0.213 | 1.237 | 1.023-1.497 | 0.028* | mRNA |
| CITED2 | 0.316 | 1.372 | 1.033-1.821 | 0.029* | mRNA |
| DNM3OS | 0.192 | 1.212 | 1.010-1.455 | 0.039* | lncRNA |
| MAP1B | 0.145 | 1.156 | 1.0051.329 | 0.043* | mRNA |
| E2F1 | -0.358 | 0.699 | 0.492-0.993 | 0.046* | mRNA |

*: *P*-value <0.05

Supplement Table 6: the prognostic differently expressed RNAs involved in ceRNA network by multivariate cox regression analysis

| gene | description | coefficient | HR | 95%CI of HR | p value |
| --- | --- | --- | --- | --- | --- |
| ERG | ETS-related gene | 0.3586 | 1.4313 | 1.142-1.793 | 0.002 |
| FASN | Fatty Acid Synthase | 0.3447 | 1.4116 | 1.097-1.817 | 0.007 |
